# Estimating the proportion of vaccine-induced hospitalized dengue cases among Dengvaxia vaccinees in the Philippines

**DOI:** 10.12688/wellcomeopenres.15507.1

**Published:** 2019-10-31

**Authors:** Stefan Flasche, Annelies Wilder-Smith, Joachim Hombach, Peter G. Smith

**Affiliations:** 1 London School of Hygiene & Tropical Medicine, London, UK; 2 World Health Organization, Geneva, Switzerland

**Keywords:** dengue, dengvaxia

## Abstract

**Background: **Dengvaxia was used in the Philippines to vaccinate 9-10-year-old school children, living in areas highly endemic for dengue. After about 830,000 had received at least 1 of 3 recommended doses, risks of enhanced disease in dengue-naïve vaccinees were reported.

**Methods: **We used Phase 3 trial data to derive the proportions of cases of hospitalised and severe dengue that might have been prevented by the Philippines vaccination programme and, among those cases that may occur in vaccinees, what proportions are likely to arise in those who were seropositive or seronegative for dengue at the time of first vaccination and what proportion in the latter group may be enhanced disease attributable to the vaccine.

**Results:** Assuming about 15% of vaccinees were dengue naïve at vaccination and the effects of the vaccine are independent of the number of doses received, we estimate that, in the 5 years following vaccination, the number of cases of severe disease in the vaccinated population will be reduced by about 70%. Among vaccinees who do develop severe disease, about half the cases will be due to vaccine breakthrough in seropositive vaccinees, and about a quarter will be excess cases in seronegative vaccinees that will have occurred as a consequence of vaccination.

**Conclusions: **Overall, the Philippine dengue vaccination programme will likely prevent a substantial number of severe dengue cases and, among those that do occur, the majority are likely to be breakthrough disease in seropositive vaccinees and a minority attributable to the excess risk of enhanced disease in seronegative vaccinees.

## Introduction

Dengue is the most frequent mosquito-borne viral disease, with a 30-fold increase in annual reported cases over the past 50 years and continued geographic expansion ^1, 2^, also affecting international travellers ^3, 4^. Infection with any of the four dengue virus serotypes may be asymptomatic or may result in clinical manifestations ranging from relatively mild febrile illness to severe dengue manifested by plasma leakage, haemorrhagic tendencies, organ failure, shock, and possibly death ^5^. Patients with a second dengue infection with a different dengue serotype to the first are at a higher risk for severe dengue than from the first infection, but subsequent infections with different serotypes are not associated with such increased risk ^5, 6^.

Dengvaxia, the first licensed dengue vaccine, is a live attenuated vaccine using the yellow fever 17D vaccine virus as its backbone. Two Phase 3 efficacy trials of Dengvaxia (then called CYD-TDV) were conducted in children aged 2–16 years in 10 countries in Asia and Latin America at sites in which dengue is highly endemic ^7, 8^. In the 25 months after the first vaccine dose, for those first vaccinated at ages 9 to 16 years, the incidence of severe dengue was 93% lower in the vaccinated group than in the placebo group and hospitalizations for virologically-confirmed dengue (VCD) were reduced by 81% ^9^. However, over the first 3 years of follow-up among those first vaccinated at ages 2–5 years, there was an overall excess of hospitalised dengue in the vaccinated group, though this excess risk was not statistically significant ^9^. At the time of first licensure of Dengvaxia in 2015, data from the Phase 3 trials did not allow assessment of whether the apparent increased risk of hospitalised and severe dengue in those first vaccinated at ages 2–5 years was related to age per se, and/or was due to a higher proportion in the younger age group being seronegative compared to older children ^10^.

Following licensure for those aged 9 years and above, an age group in which no excess risk of hospitalised or severe dengue was apparent in the trials, WHO recommended the use of the vaccine only in high transmission settings, as defined by a seroprevalence of 70% or more, to ensure substantial public health impact ^11^ To-date, Dengvaxia has only been used in two national immunization programmes, both at sub-national level, in the Philippines and Brazil ^12^.

In November 2017, Sanofi Pasteur announced the results of further analyses of data from the Phase 3 trials, using a newly developed NS1-based ELISA assay that was able to differentiate, in large part, between immune responses due to vaccine exposure and those due to natural dengue virus infection. As blood samples were taken from only a subset of trial participants prior to vaccination, but all participants had a blood sample taken one month after the third vaccine dose, the company used the results based on blood samples taken post-vaccination to infer dengue serostatus before vaccination, for those without a baseline sample. These analyses demonstrated that while the vaccine offered substantial protection among those who had been infected with dengue before vaccination, dengue-naïve vaccinees (i.e. seronegative children) were at increased risk for dengue hospitalisation and severe dengue during the 5-year trial follow-up compared to unvaccinated seronegative children ^13^. In dengue-seronegative participants aged 2–16 years, the hazard ratio (HR) for the cumulative 5-year incidence of hospitalization for VCD, comparing vaccinated to placebo recipients, was 1.75 (95% confidence interval [CI], 1.14 to 2.70), and for severe dengue the HR was 2.87 (95%CI 1.09-7.61). For those aged 9–16 years, the corresponding HRs were 1.41 (95% CI: 0.74–2.68) and 2.44 (95% CI: 0.47–12.56), respectively. In contrast, in seropositive trial participants, the vaccine was found to be efficacious and, in those aged 9–16 years, the corresponding HRs were 0.21 (95% CI: 0.14–0.31) and 0.16 (95% CI: 0.07–0.37), respectively ^13^.

Thus, in a vaccinated population consisting of a mixture of seronegative and seropositive individuals, cases of hospitalised and severe dengue that occur will be composed of breakthrough cases among those seropositive at the time of vaccination and cases among those seronegative when vaccinated. This latter group will be composed of cases that occurred because of the failure of the vaccine to protect in this group, that is, the number that would have occurred in the absence of vaccination, and induced cases, because of the enhanced risk due to vaccination in this group. The relative proportion of cases of breakthrough cases and induced cases of hospitalised and severe dengue disease will depend on the seroprevalence among those vaccinated.

Dengue is highly endemic in the Philippines and has the highest reported number of dengue cases among countries in the WHO Western Pacific region. In the first 7 months of 2019, there were 130,463 suspected dengue cases reported and 561 deaths ^14^. Following licensure of Dengvaxia in the Philippines in early 2016, there was a vaccination programme in schools in selected highly endemic regions, targeting about 1 million children aged 9–10 years. The dengue seroprevalence in this population is not known, but has been estimated to be between 80 and 85%, extrapolating from data from the trial sites in the Philippines included in the Phase 3 trial ^15^. After Sanofi Pasteur reported the safety signal in seronegative vaccinees, in November 2017, the vaccination programme was suspended. By then, over 830,000 children had received the vaccine: about 420,000 had received 1-dose, 49,000 2-doses and 370,000 3-doses (Sanofi Pasteur, personal communication). The suspension of the programme broke public trust in the dengue vaccine and heightened anxiety around vaccines in general ^16^, and cases of hospitalised and severe dengue and deaths due to dengue in vaccinated children were widely ascribed, among the public and in the media, to being vaccine-induced.

As noted above, the vaccine is only partially effective in seropositive vaccinees, so breakthrough cases would be expected in this group, and only in seronegative vaccinees would vaccine-induced cases be expected. We estimate, for those vaccinated with Dengvaxia in the Philippines, the relative proportions of hospitalized and severe dengue due to breakthrough disease in seropositive vaccinees and due to enhanced disease in seronegative vaccinees, and also estimate the proportion of hospitalized and severe cases prevented in seropositive vaccinees, extrapolating from the results from the Phase 3 trials of the vaccine.

## Methods

Our calculations are based on the retrospective analyses from the Phase 3 trials, stratified by serostatus ^13^. We used data pooled across all trial sites, which constitute a mixture of medium to high dengue burden settings, and only use data for children aged 9 years or older. Cumulative incidence estimates for dengue hospitalisations in vaccinees and controls by serological status at trial entry, for the 60 months following first vaccination, are those based on NS1 test in combination with multiple imputation techniques that also account for imperfect sensitivity and specificity of the NS1 test. These are taken from Table S10 in Sridhar *et al.* ^13^.

To infer estimates of relative numbers of dengue hospitalisations and severe dengue episodes in seropositive and seronegative vaccinees in the Philippines, we directly extrapolated from the trial data ^13^, which includes the estimated seroprevalence at trial entry, the annual incidence of dengue infections, and the relative risks of hospitalised dengue in vaccinees and controls who were seropositive or seronegative at trial entry. In brief, similar to the methods in Wilder-Smith *et al.* ^10^, a vaccinated cohort was split into dengue naïve and dengue exposed vaccinees and relative incidence estimates from the trial population were applied to each of these to obtain estimates of the expected relative number of cases among seronegative and seropositive vaccinees. We then calculate the proportion among all cases that are breakthrough cases among seropositive vaccinees, those that occur among seronegative vaccinees and the excess cases among those.

Nearly all children in the trials received 3 doses of vaccine, and it is not known if the enhanced risk of hospitalised and severe dengue in seronegative vaccinees will be the same in children who receive only 1 or 2 doses. We assumed that all vaccinees, irrespective of the number of doses received, would experience the same relative risks of hospitalized dengue and severe dengue as reported in the trial populations by Sridhar *et al*., ^13^ compared to the situation had they not been vaccinated. Hospitalisation rates for dengue varied substantially between trial sites and hence are likely not representative of the Philippines setting. Therefore, we do not report absolute incidence estimates but restrict ourselves to the reporting of relative measures which are likely to be largely unaffected by different hospitalisation rates. Based on the seroprevalence data from the trial sites in the Philippines ^15^ and further seroprevalence data in the areas where the vaccine was introduced ^17^, we assumed that the seroprevalence among those vaccinated in the Philippines was 85%.

The analyses were conducted in R version 3.5.3 and the analyses scripts can be found on github: https://github.com/StefanFlasche/Denvaxia-in-Phillippines

## Results

In the CYD-TDV Phase 3 trials, the 5-year cumulative incidences of hospitalised and severe dengue in dengue naïve vaccine recipients, aged 9–16 years at first vaccination, were increased by 44% and 132%, respectively (Sridhar *et al*, Table S10 ^13^), compared to those who were seronegative but not vaccinated. In seropositive unvaccinated children, the risks of hospitalised and severe dengue were 72% and 176% higher, respectively, than in seronegative unvaccinated children. In seropositive vaccinated children, the incidences of hospitalised and severe dengue were reduced by 80% and 84%, respectively, in the five years following vaccination compared to those who were seropositive but not vaccinated. Assuming 15% of the 830,000 children vaccinated in the Philippines were seronegative, about 125,000 are at potentially increased risk for hospitalised and severe dengue when exposed to their first natural infection with wild-type dengue virus following vaccination. Assuming that the relative risks in the Philippines are similar to those observed in the Phase 3 trials, we estimate that, over the five years following vaccination in the Philippines, Dengvaxia will have averted about 18 dengue hospitalisations among seropositive vaccinees for each precipitated dengue hospitalisation in dengue-naïve vaccinees, and about 10 severe dengue cases among seropositive vaccinees for each precipitated severe dengue case in dengue-naïve vaccinees.

**Figure 1.**
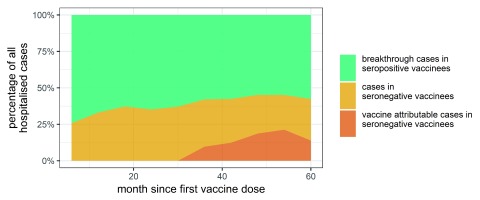
The expected percentage of hospitalised dengue cases in the Philippines vaccinated cohort that might be expected to occur in seropositive vaccinees, seronegative vaccinees and the percentage of cases in the latter group that are Dengvaxia attributable.

In the 5 years following vaccination, we estimate that, among those dengue cases that are hospitalised in the vaccinated Philippine cohort, about 58% will be vaccine breakthrough cases in seropositive vaccinees, that result from the high but imperfect effectiveness of the vaccine in seropositives ( Figure 1). We further estimate that of the 42% of hospitalised dengue cases that will occur among seronegative vaccinees, 31% of these (i.e. 13% of all hospitalised dengue cases) will be excess cases that occurred as a consequence of vaccination. However, few of those excess cases are likely to occur within 2.5 years following vaccination as the enhanced dengue risk was apparent in the trial population only from about 2 years after first vaccination ( Figure 1). In the corresponding analysis for severe dengue, the estimated proportion of cases due to vaccine breakthrough in the 5-years following first vaccination is 51%. Of the 49% of severe dengue cases that might be expected among seronegative vaccinees, 57% of these (i.e. 28% of all severe dengue cases) are excess cases that will have occurred as a consequence of vaccination.

We cannot similarly extrapolate the trial findings with respect to deaths from dengue, as there were no deaths from dengue observed in the Phase 3 trials. However, given the findings in the trials that the clinical severity of hospitalised dengue in seronegative vaccinees was similar to that in seropositive vaccinees, it seems not unreasonable to postulate that the risk of fatal outcomes would be similar, in relative terms, to those for severe dengue in seronegative and seropositive vaccinees. On this basis we speculate that, in the Philippines, in the 5-years following vaccination, for any death that might have occurred in vaccinated seronegatives around 10 deaths would be prevented by the vaccination programme in seropositives and that among all deaths from dengue in the vaccinated cohort, about 28% may be due to an enhanced risk among vaccinated seronegatives.

## Discussion

The findings from the Phase 3 trials of CYD-TDV were that, in the five years following first vaccination, the vaccine offered substantial protection against hospitalised and severe dengue among vaccinees who had been previously infected with dengue, but that in dengue-naïve vaccinees there was an increased risk of these conditions, apparent from about two years after the first vaccine dose. Thus, when the vaccine is used in public health programmes, the hospitalised and severe dengue cases that occur following vaccination will be a mixture of breakthrough cases from those who were seropositive when vaccinated and cases from those seronegative at vaccination, among whom the vaccine offered no protection and increased their likelihood of hospitalised and severe disease above the level that would have been expected had they not been vaccinated. The relative proportions of cases arising through these different mechanisms will depend upon the dengue-seroprevalence among those vaccinated. We have tried to estimate these proportions, among children who were included in the mass vaccination programme with Dengvaxia in the Philippines, based on the assumption that about 85% of the children vaccinated would have had a previous dengue infection before they were vaccinated. The first point to note is that among all those vaccinated, the total number of hospitalised and severe dengue cases in the five years following vaccination would be expected to be reduced by 69% and 71%, respectively, compared to the situation had no children been vaccinated. Among the cases that would be expected to occur in the vaccinated population, we estimate that more than 50% of the cases would be in vaccinees who were seropositive at the time of vaccination, as a consequence of breakthrough disease. Among seronegative vaccinees, a sizeable amount of cases of hospitalised and severe dengue also would have occurred in the absence of vaccination, but about 13% of all hospitalised cases and of all 28% of severe cases would be excess cases attributable to the enhanced risk associated with the vaccination of seronegatives.

It is important to note some of the limitations in our estimates. We have extrapolated from the results from the Phase 3 trials directly to the public vaccination programme in the Philippines. In particular, we have assumed that the relative risks were the same for children who were partially vaccinated as for those who received all 3 doses. We have no data for partially vaccinated children in the Phase 3 trials as all virtually received 3 doses, and thus do not know if partial vaccination alters the risk of enhanced disease in seronegative vaccinees or reduces the protective effect in seropositive vaccinees. In the phase 3 trials, the overall efficacy between the first and second, and second and third vaccine doses were similar and the immunogenicity in seropositives was similar after 1, 2 or 3 doses ^18^. The majority of children in the Philippines received only 1-dose and only about 44% received all 3 doses. Further, we assume that 85% of vaccinees will have been seropositive when vaccinated, resulting in an estimated 13% of 5-year cumulative dengue hospitalisations in the vaccinated cohort being attributable to excess cases among seronegative vaccinees. If, instead, we assume that 70% or 90% of vaccinees were seropositive, then, the estimated excess cumulative hospitalisations attributable to excess cases among seronegative vaccinees would be 20% or 10%, respectively, rather than 13%. Other limitations include that we do not account for differential vaccine effectiveness by serotype, do not extend our predictions beyond the 5-year observation period of the trial and do not account for more complex immunological dynamics potentially arising from differences in population immunity predating vaccination between the trial population and the vaccinated cohort in the Philippines. In addition, media publicity around Dengvaxia in the Philippines may have led to milder cases of dengue among vaccinated children being hospitalised and thus, extrapolation based on hospitalised cases in the Phase 3 trials may not be valid, though it should be noted that there was substantial variation between different sites in the Phase 3 trials in the proportion of all dengue cases that were hospitalised ^7, 9, 19^, presumably reflecting variation in admission practices for dengue.

We also restricted our analyses to inference based on point estimates of the relative disease rates in the trials. However, uncertainty in all point estimates is substantial. We have chosen this approach because the reported confidence intervals on incidence and relative risk in the trials are likely highly correlated and hence using the published data it is not possible to reflect uncertainty adequately in the analyses.

Perhaps most uncertain are the results we have inferred with respect to deaths from dengue. During the clinical trials, no deaths from dengue were reported. Outside of the special circumstances of a trial, the case fatality ratio of hospitalised dengue will likely be higher. Furthermore, we have assumed that the findings in the trials with respect to the relative risks of severe dengue will approximate those for deaths from dengue. We cannot validate this assumption, though to us it seems plausible.

Our results cannot be used for causality assessment with respect to individual dengue cases. Only a proportion of hospitalised and severe dengue cases occurring in individuals without previous dengue infection at the time of vaccination would be related to an enhanced risk associated with vaccination. Clinical management of severe dengue is the same, irrespective of baseline serostatus at time of administration of Dengvaxia. Testing already vaccinated children with the NS1 based IgG ELISA would not change clinical management. Furthermore, this test has imperfect sensitivity and specificity ^20^. Testing all vaccinees to infer retrospectively their serostatus at the time of vaccination, using for example the NS1 test, may provide false assurance in those estimated to be seropositive at the time of vaccination, as they are still at risk of hospitalised or severe dengue due to breakthrough disease.

In summary, our calculations may help estimate the respective distribution of severe dengue cases among presumably seropositive and seronegative individuals following vaccination. They indicate that in high transmission settings a majority of hospitalised and severe dengue cases among vaccinees are expected to come from subjects presumably seropositive at time of vaccination. The relative number of cases with breakthrough dengue and enhanced dengue will depend on the seroprevalence among those vaccinated.

Dengvaxia still has a potential public health role, in the absence of currently available alternative solutions, to combat the expanding problem of the global dengue burden. The challenge is how best to use the vaccine balancing the potential for public health impact in those previously infected with dengue, against the possibility of harm in dengue-naïve vaccinees. In communities in which the prevalence of past infection with dengue is high in the group targeted for vaccination, and in the absence of the application of a specific test to identify previously infected persons, the risk of severe dengue will be, on average, higher in persons not vaccinated than persons vaccinated ^10^. The protective effect in the majority who are seropositive being greater than the enhancing effect in the minority who are seronegative. However, in the latter group some will develop severe dengue who would not have developed this in the absence of vaccination. This poses substantial communication challenges for those responsible for vaccination programmes. WHO has recommended that for countries considering vaccination as part of their dengue control programme, a “pre-vaccination screening strategy” is recommended, in which only dengue-seropositive persons are vaccinated ^21^. However, this strategy also presents major challenges in terms of logistics, implementation, communication and additional costs, as well as the current absence of a sensitive and specific rapid point-of-care test to assess serostatus ^22^.

## Data availability

### Underlying data

Underlying data on the estimated incidence of dengue hospitalisations in the trials is available from Shridar *et al*. ^13^ and digitalised in the analysis script at https://github.com/StefanFlasche/Denvaxia-in-Phillippines

### Extended data

Scripts used for analysis available from: https://github.com/StefanFlasche/Denvaxia-in-Phillippines

Archived scripts as at time of publication: http://doi.org/10.5281/zenodo.3496820 ^23^

Data are available under the terms of the Creative Commons Zero “No rights reserved” data waiver (CC0 1.0 Public domain dedication).
