# Supplementary material for: Estimating the proportion of vaccine-induced hospitalized dengue cases among Dengvaxia vaccinees in the Philippines
Source: Wellcome Open Res. 2019 Oct 31;4:165. [Version 1] doi: 10.12688/wellcomeopenres.15507.1 (PMC6880258; doi:10.12688/wellcomeopenres.15507.1)
Supplement: Peer review history

# Peer review: Reviewer response for version 1

**DOI:** 10.21956/wellcomeopenres.16972.r36915

**Published:** 2019-11-26

**Authors:** Quirine A. ten Bosch

**Affiliations:** 1 Wageningen University & Research, Wageningen, The Netherlands

While Dengvaxia, the first licensed dengue vaccine, is shown to illicit effective protection in individuals pre-exposed with dengue, increased risks of hospitalization in seronegative individuals were demonstrated in retrospective analyses. This has resulted in the suspension of the vaccination program in the Philippines. The WHO has altered its recommendation on the use of Dengvaxia. Use should be restricted to highly endemic areas (seroprevalence >70%), exclude the youngest age groups, and pre-vaccination screening should be incorporated into the strategy.

 The mixed outcomes of the vaccine have raised questions on its population-level impact. In this paper, the authors extrapolate estimates from the Phase-3 dengvaxia trial to estimate, among observed vaccinated severe cases/ hospitalizations, what proportion is attributable to vaccine failure vs vaccine induced morbidity. In addition, they estimated the number of cases averted per vaccine induced case. They conclude that, in a setting with high enough seroprevalence, the number of averted cases would outnumber the number of cases attributable to excess risk of enhanced disease in seronegative vaccines.

 The work is timely and well performed and limitations are adequately described. I do have a few comments, in particular about the description of the methods and presentation of results.

**Methods**

- The methods are quite densely described and become more clear once referring to the (well-documented!) code. The paper would benefit from a more detail in the methods, including the relevant equations.
- Further, from the methods itself it is not clear how the authors derive the time-dependent estimates as shown in figure 1.

**Results**

- Figure 1 is nice, but does not show the full picture given that the incidence increases over time. Therefore, the larger proportion of cases attributable to the vaccine in seronegative cases towards the end of the follow-up contributes more to the overall number of cases. A cumulative proportion might be more informative.
- It is not always clear what is meant with seroprevalence. If this refers to the seroprevalence in the population rather than the 9-16 year-olds, the seroprevalence in the code should be adjusted to reflect the seroprevalence expected in that age-group.  
- The authors report on the sensitivity of their results for settings with higher and low seroprevalence. This is a big uncertainty and a hurdle for implementation. Particularly since local, age-specific seroprevalence is hard to obtain. It would therefore be informative to see a bit larger sweep across seroprevalences, particularly for the number of hospitalized cases averted per vaccine-induced case and how this varies across endemicities. 
- Lastly, a small textual remark: it is not clear to me why only vaccine failure in seropositives is considered a breakthrough infection.

I confirm that I have read this submission and believe that I have an appropriate level of expertise to confirm that it is of an acceptable scientific standard.

# Peer review: Reviewer response for version 1

**DOI:** 10.21956/wellcomeopenres.16972.r36916

**Published:** 2019-11-18

**Authors:** Stephen H. Waterman, Talia Quandelacy

**Affiliations:** 1 Dengue Branch, Centers for Disease Control and Prevention, San Juan, Puerto Rico; 2 Dengue Branch, Centers for Disease Control and Prevention, San Juan, PR, USA

In the aftermath of the Philippines dengue vaccine controversy, the authors contribute an analysis of the expected dengue hospitalizations and severe illnesses among the approximately 800,000 9-10 year-old school children who participated in a vaccination program with the newly licensed vaccine during 2016 and 2017 in highly dengue endemic areas of the Philippines.

 In November 2017 Sanofi Pasteur reported that dengue seronegative children of all ages vaccinated with CYD-TDV (Dengvaxia) had higher risk of hospitalization (1.75, 1.14-2.70, reference 13)) and severe dengue illness, while the vaccine considerably reduced such risk in seropositive children (0.32, 0.23-0.45). This announcement at the time of government turnover precipitated the Philippines controversy and an end to the program. The new data resulted in updated WHO Strategic Advisory Group of Experts (SAGE) recommendations for CYD-TDV emphasizing a preferred approach of pre-vaccination screening for prior dengue infection before vaccination.

 The authors, all members of the WHO SAGE committee making recommendations on CYD-TDV, estimate the proportions of hospitalized and severe cases among vaccinated Philippine children using the Sanofi Phase 3 trial data for children 9 years and older. The calculations make use of published virologically confirmed hospitalization incidence and hazard ratios in seropositive and seronegatives including supplemental data from the clinical trials over five years post vaccination series completion, and an empirically realistic assumption of 85% seroprevalence in the 9-10 year olds in the Philippine sites.

 The results clarify that the majority of hospitalizations and severe illnesses in vaccinated children are likely due to CYD-TDV vaccine breakthrough in seropositive children, while excess cases associated in vaccinated seronegative children would comprise about a quarter of all such cases. Another 25 percent of hospitalizations and severe illnesses would have occurred in seronegative children regardless of vaccination. With the seroprevalence assumption of 85%, the vaccination program would avert 18 hospitalizations for each excess hospitalization in seronegative vaccinees. Using the same proportions as severe cases, the authors also estimate that 10 deaths would be prevented for each death that might have occurred in vaccinated seronegatives.

 The methods calculations are standard epidemiologic measures (i.e., excess risk, population attributable fractions, and cases averted), and data and code for the generating the estimates are conveniently placed in a github link for accessibility. The discussion appropriately includes a sensitivity analysis using seroprevalences of 70% and 90% since dengue seroprevalence has a significant effect on the estimates. However, some aspects of the methods could be expanded to improve reader accessibility.

 The manuscript is well written and provides a useful perspective on the difficult Philippines situation. The figure is quite helpful in conveying the proportion and timing of all hospitalized cases attributable to vaccinating seronegatives. The authors clearly state the background for the study and the limitations of the analysis, including that this analysis cannot address individual causality for severe illness. The manuscript concludes with an appropriate summary of the need for public health decision makers to balance the potential population benefits of CYD-TDV with challenges of logistics and specificity of pre-vaccination screening, and communications to the public and medical communities of the relative benefits and risk.

 Comments

 Introduction

- Given the reference to vaccine breakthrough in the methods and results, providing summary estimates of CYD-TDV vaccine efficacy would be helpful to the reader.

Methods

- Little numerical data are provided in the methods. A table or figure of the trial populations would be helpful.
- While the code with the formulas and calculations is on the github, providing the formulas for the cases averted and excess cases in the text would be useful to understand the estimates.

Results

- That the R code on the Github makes calculations with 70% seroprevalence when the paper presents results based on the assumed 85% seroprevalence is somewhat confusing.

Discussion

- Page 6, first paragraph: The authors indicate they did not provide confidence intervals for the point estimates of hospitalizations and severe illnesses, which as stated have substantial uncertainty, because of incertain uncertainty arising from likely correlation of trial incidence and relative risk confidence intervals. As the first author has published on mathematical models of the Sanofi trial (PLoS Med. 2016 ^1^), it seems reasonable that the paper could provide confidence intervals for the point estimates.

We confirm that we have read this submission and believe that we have an appropriate level of expertise to confirm that it is of an acceptable scientific standard.

## References

[1] : The Long-Term Safety, Public Health Impact, and Cost-Effectiveness of Routine Vaccination with a Recombinant, Live-Attenuated Dengue Vaccine (Dengvaxia): A Model Comparison Study. *PLoS Med*.2016;13(11) : 10.1371/journal.pmed.1002181 e1002181 10.1371/journal.pmed.1002181 27898668PMC5127514
